# Role of MicroRNA-214 in Dishevelled1-Modulated β-catenin Signalling in Non-Small Cell Lung Cancer Progression

**DOI:** 10.7150/jca.80291

**Published:** 2023-01-01

**Authors:** Huanyu Zhao, Zhao Wang, Guangping Wu, Yudie Lu, Jingrong Zheng, Yue Zhao, Yang Han, Jian Wang, Lianhe Yang, Jiang Du, Enhua Wang

**Affiliations:** 1 Department of Pathology, The First Hospital and College of Basic Medical Sciences, China Medical University, Shenyang, China.; 2 Department of Pathology, Beidahuang Industry Group General Hospital, Harbin, Heilongjiang, China.

**Keywords:** Dishevelled1, microRNA-214, CTNNB1, non-small cell lung cancer, coexpression

## Abstract

**Background:** The mortality of patients with non-small cell lung cancer (NSCLC) is rather high. This is largely because of the lack of specific targets and understanding of the molecular mechanism for early diagnosis. Dishevelled (Dvl) dysregulation leads to malignant progression. We confirmed that Dvl1 expression is associated with a poor prognosis of patients with NSCLC. However, how Dvl1 transmits signals through the Wnt/β-catenin pathway remains unknown.

**Methods:** In this study, the expression levels of Dvl1 and β-catenin in resected NSCLC samples were immunohistochemically analysed. Dvl1 cDNA and small interfering RNA against β-catenin were transfected into NSCLC cells, and their effects on canonical Wnt signalling and biological behaviour of NSCLC cells were analysed. Using bioinformatics analyses, an interaction between microRNA (miR)-214 and β-catenin was identified; miR-214 expression was determined in NSCLC tissues using quantitative real-time polymerase chain reaction. An exogenous miR-214 (mimic) was used to analyse the biological behaviour of NSCLC cells and the effect of Dvl1 on canonical Wnt activation.

**Results:** Dvl1 overexpression in NSCLC tissues as well as Dvl1 and β-catenin nuclear coexpression were significantly associated with poor prognosis of NSCLC (*P* < 0.05). Additionally, Dvl1 promoted Wnt/β-catenin signalling to enhance the malignant phenotype of NSCLC cells. Moreover, miR-214 directly targeted the 3′ untranslated region of β-catenin to inhibit the activation of canonical Wnt signalling induced by Dvl1.

**Conclusions:** Our results suggest that Dvl1 is a potential therapeutic target for NSCLC and that miR-214 plays an inhibitory role in Dvl1-mediated activation of Wnt/β-catenin signalling in NSCLC cells, which could affect NSCLC progression.

## Introduction

Lung cancer is a serious health problem worldwide 1. Non-small cell lung cancer (NSCLC) accounts for more than 85% of all lung malignancies 2. Despite the availability of many therapeutic approaches, the mortality rate of NSCLC is rather high. This is largely due to the lack of specific targets and understanding of the detailed underlying molecular mechanisms. Therefore, the exploration of effective biomarkers for early diagnosis is important for improving the prognosis of NSCLC.

MicroRNAs (miRNAs, miRs) are highly conserved, noncoding small RNAs that are 18-24-nucleotide long and play important regulatory roles in tumorigenesis. They interact with complementary target mRNAs to inhibit their translation. Several miRNAs, including miR-214 3, play inhibitory roles in canonical Wnt/β-catenin activation 4.

Wnt signalling regulates normal cellular development, but its dysregulation promotes tumorigenesis 5. It is activated by Wnt ligands, and the initiated intracellular signalling cascades transduce signals to two main branches: the canonical and noncanonical Wnt pathways. Canonical Wnt/β-catenin signalling promotes the nuclear translocation of β-catenin to activate lymphoid enhancer factor (LEF) and T-cell factor (TCF), which are transcription factors involved in malignant progression 6. The abnormal activation of Wnt signalling is prominent in human malignancies. Sustained canonical Wnt signalling activation is common in NSCLC, and many Wnt target genes are altered 7.

Dishevelled (Dvl), belonging to a family of phosphoproteins, relays signals that arise from all Wnt ligands to mediate tumour growth 8. Three isoforms of Dvl—Dvl1, Dvl2, and Dvl3—are encoded by mammalian genes. Dvl1 has been studied extensively because it is a crucial component of the canonical Wnt/β-catenin pathway 9. Dvl1 can activate Wnt/β-catenin signalling in NSCLC, which is overexpressed in patients with NSCLC, and is associated with poor prognosis 10. However, the mechanism by which Dvl1 regulates Wnt/β-catenin signalling is poorly understood.

In this study, using TargetScan target prediction, we identified a potential interaction between miR-214 and the 3′-untranslated region (UTR) of β-catenin and elucidated the role of miR-214 in Dvl1-modulated Wnt/β-catenin signalling. The findings of this study might be valuable in the identification of new therapeutic targets for NSCLC.

## Materials and Methods

### Tissue specimens

Twenty-four fresh NSCLC samples from patients who had undergone surgery were selected from the First Hospital of China Medical University in 2017. We excluded chemotherapy- and radiotherapy-treated patients. Corresponding paracancerous tissues (at least 5 cm from the lesion) were collected for comparison. The samples were frozen in liquid nitrogen and stored at -80 °C for quantitative real-time polymerase chain reaction (qRT-PCR) analysis.

Tumour samples from 122 patients with NSCLC, who underwent surgical resection but did not undergo chemotherapy or radiotherapy prior to surgery, at the First Hospital of China Medical University between 2013 and 2015, were selected for immunohistochemical analysis.

This study was conducted under the guidelines of the Institutional Review Board of China Medical University (No. 2016 [LS] 014). Written informed consent was obtained from all patients.

### Immunohistochemistry

Immunohistochemical procedures were the same as previously described 11. Primary antibodies against Dvl1 (1:100; sc-8025, Santa Cruz Biotechnology, Dallas, TX, USA) and β-catenin (1:200; 8480, Cell Signaling Technology, Danvers, MA, USA) were used in the study. Score evaluation was based on the following criteria: (a) Dvl1 positivity was defined by ≥ 10% positively stained cancer cells; and (b) β-catenin expression was classified as either normal (≥ 80% cancer cells with positive membranous staining) or abnormal (≥ 10% cancer cells with positive nuclear or cytoplasmic staining).

### Cell culture

The human NSCLC cell lines H1299, SPC, A549, and H157 and the human bronchial epithelial cell line HBE were obtained from the American Type Culture Collection (Manassas, VA, USA). Short tandem repeat (STR) analysis was performed to test the authenticity of the cell lines. We selected A549 and H157, which have relatively low Dvl1 expression (Figure S1), for this study. The cells were cultured in Dulbecco's modified Eagle's medium (Thermo Fisher Scientific, Waltham, MA, USA) containing 10% foetal bovine serum (Gibco, Carlsbad, CA, USA) and 1% penicillin/streptomycin (Sigma-Aldrich, St Louis, MO, USA) in 5% CO_2_ at 37 °C.

### qRT-PCR

Total RNA was extracted from NSCLC tissue specimens, paracancerous tissues, and NSCLC cell lines using TRIzol (Invitrogen, Carlsbad, CA, USA). qRT-PCR was performed as described previously 12. The primer sequences used in this study are listed in Table S1.

### Reagents and transfection

The 3′-UTR of the β-catenin mRNA *CTNNB1* (accession no. NM_001098209.1) was amplified using PCR and subcloned into the pGL3 vector MCS cloning site (Promega Corporation, Madison, WI, USA). *CTNNB1* was classified as either wild type (WT) or mutant (Mut, binding sites for miR-214), and pGL3-WT-CTNNB1-3′-UTR and pGL3-Mut-CTNNB1-3′-UTR plasmids were generated. The sequence for the *CTNNB1* 3′-UTR forward primer was 5′-TCTAGAATACAATGACTT TTTAGCTG-3′ and that for the reverse primer was 5′-TCTAGATTAGCCAAG-3′.

miR-214 mimic (sense, 5′-ACAGCAGGCACAGACAGGCAGU-3′ and antisense, 5′-UGCCUGUCUGUGCCUGCUGUUU-3′), mimic negative control (sense, 5′-UUCUCCGAACGUGUCACGUTT-3′ and antisense, 5′-ACGUGACACGUUCGGAGAATT-3′), miR-214 inhibitor (5′-UGCCUGUCUGUGCCUGCUGUUU-3′), inhibitor negative control (5′-CAGUACUUUUGUGUAGUACAA-3′), and small interfering (si)RNA-β-catenin were obtained from Shanghai GenePharma Co., Ltd. (Shanghai, China). The Dvl1 expression vector pCS2-Myc-Dvl1 was provided by Dr. Zhenge Luo (Institute of Neuroscience, Shanghai Institutes of Biological Sciences, Chinese Academy of Sciences). NSCLC cells were transfected with the above plasmids or miRNAs (final concentration: 20 nM) using Lipofectamine 2000 (Thermo Fisher). Transfected cells were incubated for 48 h at 4 °C for further experiments.

### Western blotting

Total protein extracted from NSCLC cells (50 μg) was quantified using the Bradford method and then separated using sodium dodecyl sulfate-polyacrylamide gel electrophoresis. After being transferred to polyvinylidene fluoride membranes, the membranes were blocked with 5% skim milk for 2 h at room temperature (24-26 °C) and then incubated overnight (4 °C) with primary antibodies against one of the following: c-Myc (#5605, 1:500; Cell Signaling Technology), Cyclin D1 (#55506,1:500; Cell Signaling Technology), β-catenin (#8480, 1:500; Cell Signaling Technology), GAPDH (#5174, 1:500; Cell Signaling Technology), Myc tag (R951-25, 1:500, Thermo Fisher), laminB1 (sc-377000, 1:500, Santa Cruz), and α-tubulin (sc-8035, 1:500, Santa Cruz). On the second day, we incubated the membranes with a horseradish peroxidase-labelled secondary antibody (Santa Cruz) for 2 h at room temperature. Subsequently, a BioImaging System (UVP Inc., Upland, CA, USA) was used to analyse the protein bands densitometrically.

### Immunofluorescence

The cells were fixed with 4% paraformaldehyde and incubated with primary antibodies against either Dvl1 (1:50; Santa Cruz) or β-catenin (1:100; Cell Signaling Technology) at 4 °C overnight and then incubated with goat anti-mouse IgG-FITC (OriGene, Rockville, MD, USA) at room temperature (24-26 °C) in the dark. We counterstained the nuclei with Hoechst 33258 (Thermo Fisher). The images were acquired using a confocal laser scanning microscope (Olympus, Japan).

### Luciferase reporter assay

NSCLC cells were seeded in 6-well plates (5 × 10^5^ cells/well) and then treated under specific conditions. We transiently co-transfected NSCLC cells with the TCF/LEF firefly luciferase construct (100 ng) and the *Renilla* luciferase vector (10 ng) (Yeasen Biotech Co., Ltd., Shanghai, China). After incubation for 24 h at room temperature, luciferase activity was analysed using a Dual-Luciferase Reporter Assay System (Promega Corporation).

### Transwell assay

The procedures and experimental equipment used for the Transwell assay have been described previously 12.

### Bioinformatics method

To predict the potential upstream miRNAs of *CTNNB1*, TargetScan (http://www.targetscan.org/vert_72/) was used. Pearson correlation analysis was performed between the potential candidate miRNAs and *CTNNB1*.

### Statistical analysis

GraphPad 5.0 software (GraphPad, San Diego, CA, USA) was used for statistical analysis. Results from at least three independent experiments were presented as mean ± standard deviation (SD). The correlation between miR-214 and β-catenin was analysed using Pearson's correlation analysis. Differences between two groups were assayed using Student's *t*-test. Significant multiple-group differences were analysed using one-way analysis of variance (ANOVA). *P* < 0.05 was considered to indicate statistical significance.

## Results

### High Dvl1 expression in NSCLC tissues is significantly associated with a poor prognosis in patients with NSCLC

**Figure 1:**
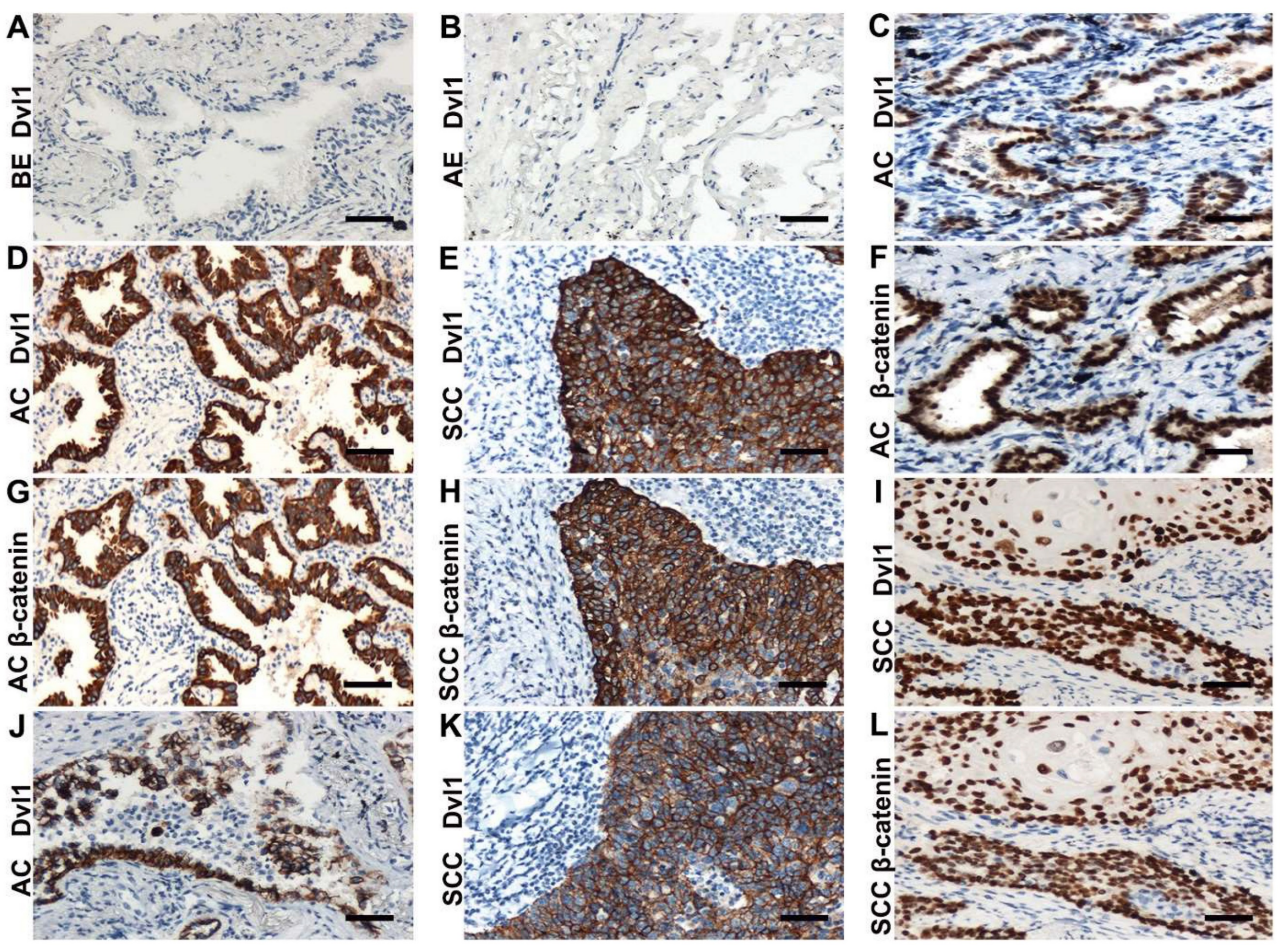
Immunohistochemical examination. (A) Dvl1 negative immunostaining in the bronchial epithelium (BE) (B) and alveolar epithelium (AE); (C, F) Dvl1 and β-catenin nuclear positive staining in adenocarcinoma (AC); (D, G) Dvl1 and β-catenin cytoplasmic positive staining in AC; (E, H) Dvl1 and β-catenin cytoplasmic positive staining in squamous cell carcinoma (SCC); (I, L) Dvl1 and β-catenin nuclear positive staining in SCC; (J, K) Dvl1 membranous positive staining in AC and SCC (A-L, scale bar = 70 μm).

Immunohistochemistry was applied to analyse Dvl1 expression in 122 NSCLC and 32 normal lung tissue samples. Bronchial and alveolar epithelial cells from normal lung tissue samples showed negative Dvl1 expression (Figure 1A, B). Positive Dvl1 expression was observed in 75 cases (61.5%) of NSCLC. In addition, *Dvl1* mRNA levels were significantly higher in NSCLC tissues than in paracancerous tissues (Figure S2, *P* < 0.05).

**Figure 2:**
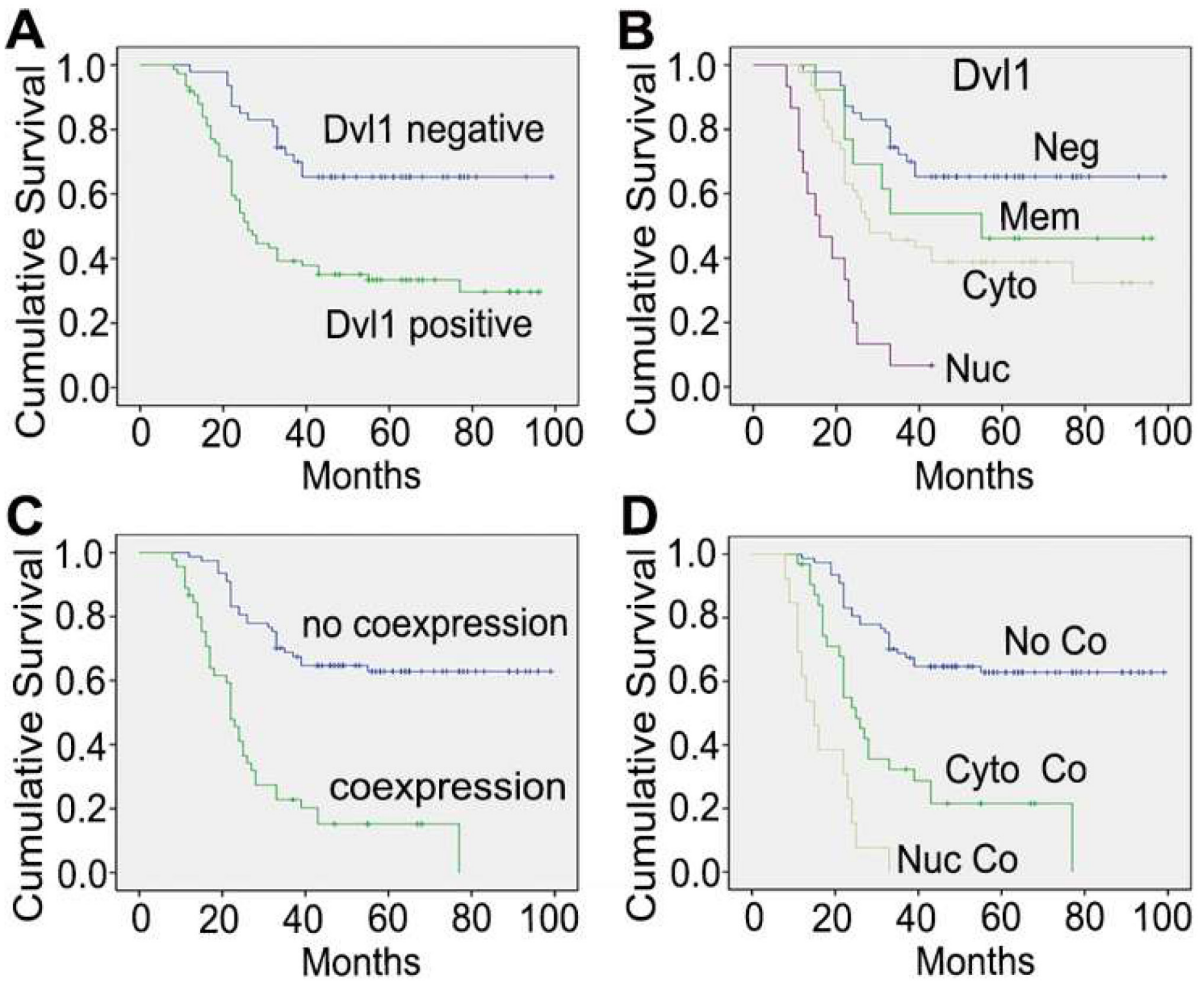
Kaplan-Meier curves of overall survival among different patients. (A) The groups with Dvl1 positive expression and negative expression; (B) the groups with Dvl1 negative expression (Neg), positive membranous expression (Mem), positive cytoplasmic expression (Cyto), and positive nuclear expression (Nuc); (C) the groups with Dvl1 and β-catenin coexpression and non-coexpression in the nucleus and cytoplasm; (D) the groups with Dvl1 and β-catenin nuclear coexpression (Nuc Co), cytoplasmic coexpression (Cyto Co), and non-coexpression (No Co).

In the NSCLC group with Dvl1 positivity, nuclear (Figure 1C, I), cytoplasmic (Figure 1D, E), and membranous (Figure 1J, K) expression was observed. The correlation between Dvl1 overexpression and clinicopathological characteristics is shown in Table 1. Dvl1 expression was positively correlated with tumour differentiation (*P* < 0.05) but not significantly associated with sex, age, or lymphatic metastasis (*P* > 0.05). Dvl1 positivity was considerably higher in lung adenocarcinomas than in lung squamous cell carcinomas and significantly higher in stages II-III than in stage I (*P* < 0.05). Kaplan-Meier analysis suggested a notably lower survival period for patients with NSCLC with positive Dvl1 expression than for patients with negative Dvl1 expression (74.486 ± 5.000 vs 46.334 ± 4.085 months, *P* < 0.05; Figure 2A).

### Dvl1 and β-catenin nuclear coexpression is significantly associated with poor prognosis of NSCLC

Dvl1 was expressed in different locations in NSCLC tissues, corresponding to different prognoses (Figure 2B). Compared to the group with negativity and membranous positivity, the group with cytoplasmic or nuclear positivity had relatively poor prognoses. β-catenin positivity was considerably correlated with poor prognosis in NSCLC (Figure S3A). It was expressed in different locations (Figure 1F-H, L), corresponding to different prognoses (Figure S3B). A significant positive correlation was found between Dvl1 and β-catenin overexpression in NSCLC tissues (*P* < 0.05, Table 1).

The coexpression rates for Dvl1 and β-catenin were 68.1% (32/47, Table 2) in the cytoplasm and 86.7% (13/15, Table 2) in the nucleus (Figure 1C-I, L). Their expression was significantly correlated in both the cytoplasm (Table 2, *P* < 0.05, *r* = 0.339) and nucleus (Table 2, *P* < 0.05, *r* = 0.848). The coexpression rate for β-catenin and Dvl1 in the membrane was 15.4% (2/13, Table 2); their membranous coexpression was not correlated (*P* > 0.05; Table 2).

Kaplan-Meier analysis showed a significantly lower survival period in patients with cytoplasmic and nuclear coexpression of Dvl1 and β-catenin than in patients without coexpression (29.914 ± 3.319 vs 72.447 ± 4.027 months, *P* < 0.05; Figure 2C). The survival period for the nuclear coexpression group was considerably lower than that of the cytoplasmic coexpression group (17.077 ± 2.108 vs 35.226 ± 4.298 months, *P* < 0.05; Figure 2D). Therefore, cytoplasmic or nuclear coexpression of Dvl1 and β-catenin, especially nuclear coexpression, was significantly correlated with poor prognosis of NSCLC.

### Dvl1 promotes Wnt/β-catenin signalling to enhance the malignant phenotype of NSCLC cells

**Figure 3:**
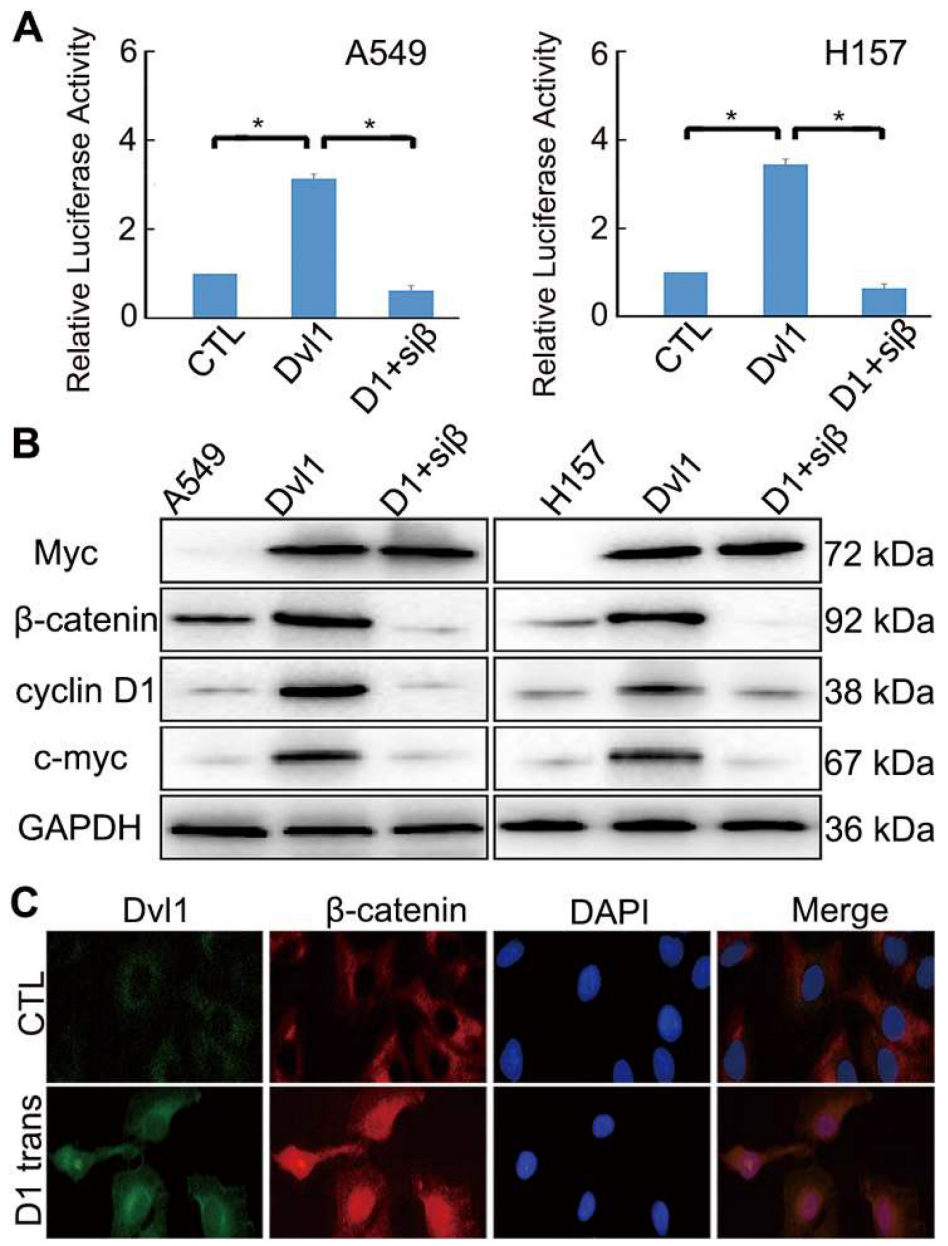
Impact of Dvl1 on the Wnt/β-catenin pathway. (A) Dual-luciferase assay analyses of Wnt/β-catenin signalling. (B) Western blotting analyses of target proteins; transfection of Dvl1 expression plasmid (Dvl1) or co-transfection of siRNA-β-catenin with Dvl1 (D1+siβ) into NSCLC cells. (C) Analysis of Dvl1 and β-catenin localisation by confocal microscopy (magnification: ×400); transfection of Dvl1 expression plasmid (D1 trans) to A549 cell. CTL, transfected negative control plasmid of Dvl1 expression plasmid. *, *P* < 0.05.

A549 and H157 cells showed relatively low Dvl1 expression (Figure S1). A549 and H157 cells were transfected with Myc-tagged-Dvl1 expression plasmids. β-catenin-dependent TCF transcription activity was significantly enhanced (*P* < 0.05; Figure 3A). The target gene expression of the Wnt/β-catenin pathway,* c-Myc,* and *CCND1* (which encodes Cyclin D1) was significantly upregulated (*P* < 0.05; Figure 3B). Moreover, immunofluorescence analysis revealed that Dvl1 transfection promoted β-catenin nuclear translocation (Figure 3C).

Nuclear translocation of β-catenin promotes its interaction with TCF, resulting in the activation of downstream target genes 13,14. Adding siRNA-β-catenin significantly inhibited Dvl1-induced TCF transcription activity and Cyclin D1 and c-Myc expression (*P* < 0.05; Figure 3A, B).

**Figure 4:**
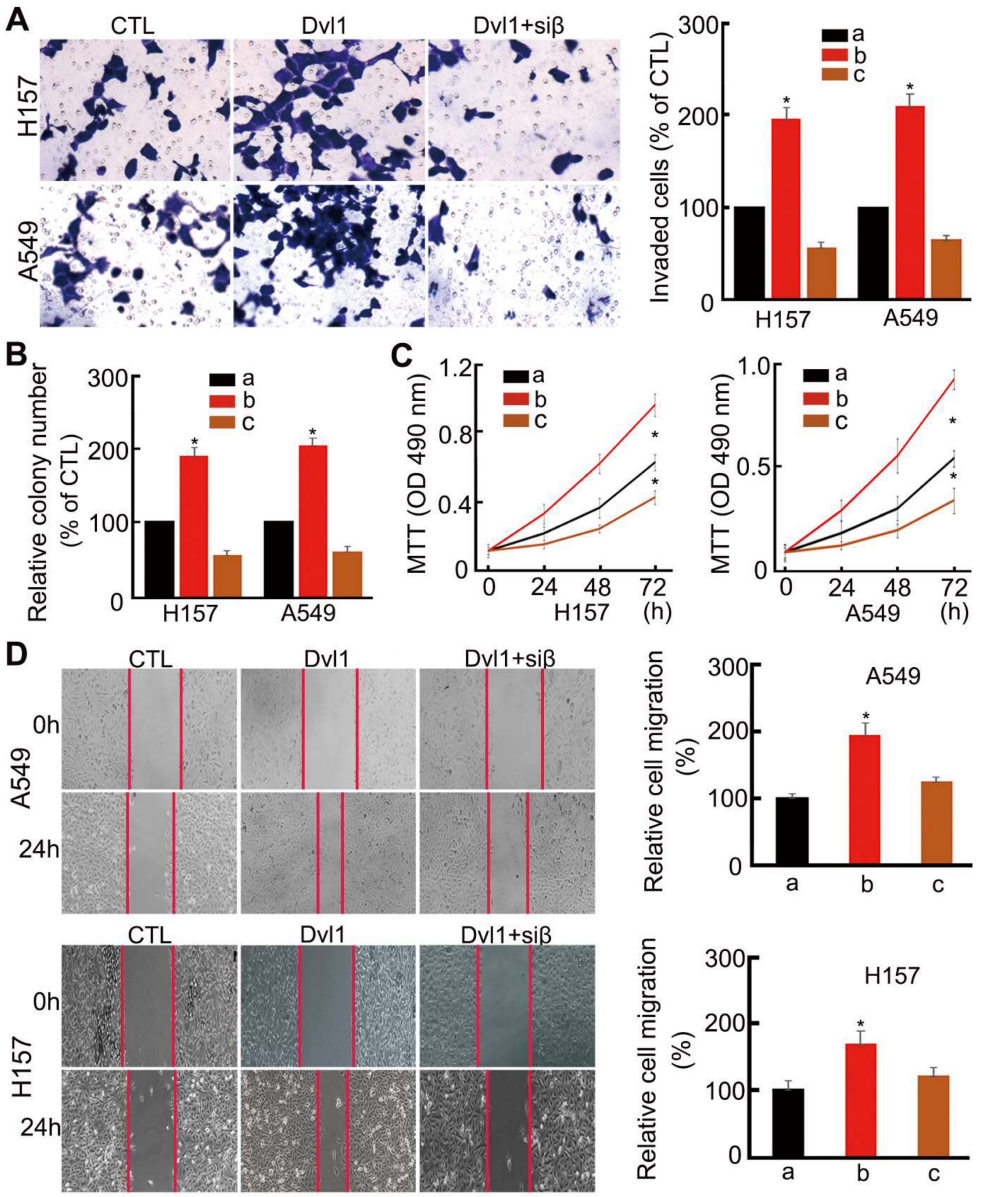
Influence of Dvl1 on the biological behaviour of NSCLC cells. (A) Invasiveness, (B) colony formation, (C) proliferation, and (D) migration of A549 and H157 cells; Dvl1, transfection of Dvl1 expression plasmid; CTL, negative control group of Dvl1 expression plasmid. Siβ, siRNA-β-catenin. The graphs show cell (A) invasiveness and (D) cell migration under different treatments; a, CTL; b, transfection of Dvl1 expression plasmid; c, co-transfection of Dvl1 expression plasmid and siRNA-β-catenin. *, *P* < 0.05.

Analysis of cell biological behaviour confirmed that NSCLC cell invasiveness, migration, colony formation, and proliferation were significantly enhanced by Dvl1 transfection (*P* < 0.05). After adding siRNA-β-catenin, Dvl1-induced biological behaviours, including invasion, proliferation, and migration, were significantly inhibited (*P* < 0.05; Figure 4).

### miR-214 directly targets β-catenin to inhibit Wnt/β-catenin signalling in NSCLC cells

β-catenin is a target of miR-214 15. Bioinformatics analysis identified a potential miR-214-binding site in the 3′-UTR of the β-catenin gene *CTNNB1* (Figure S4).

**Figure 5:**
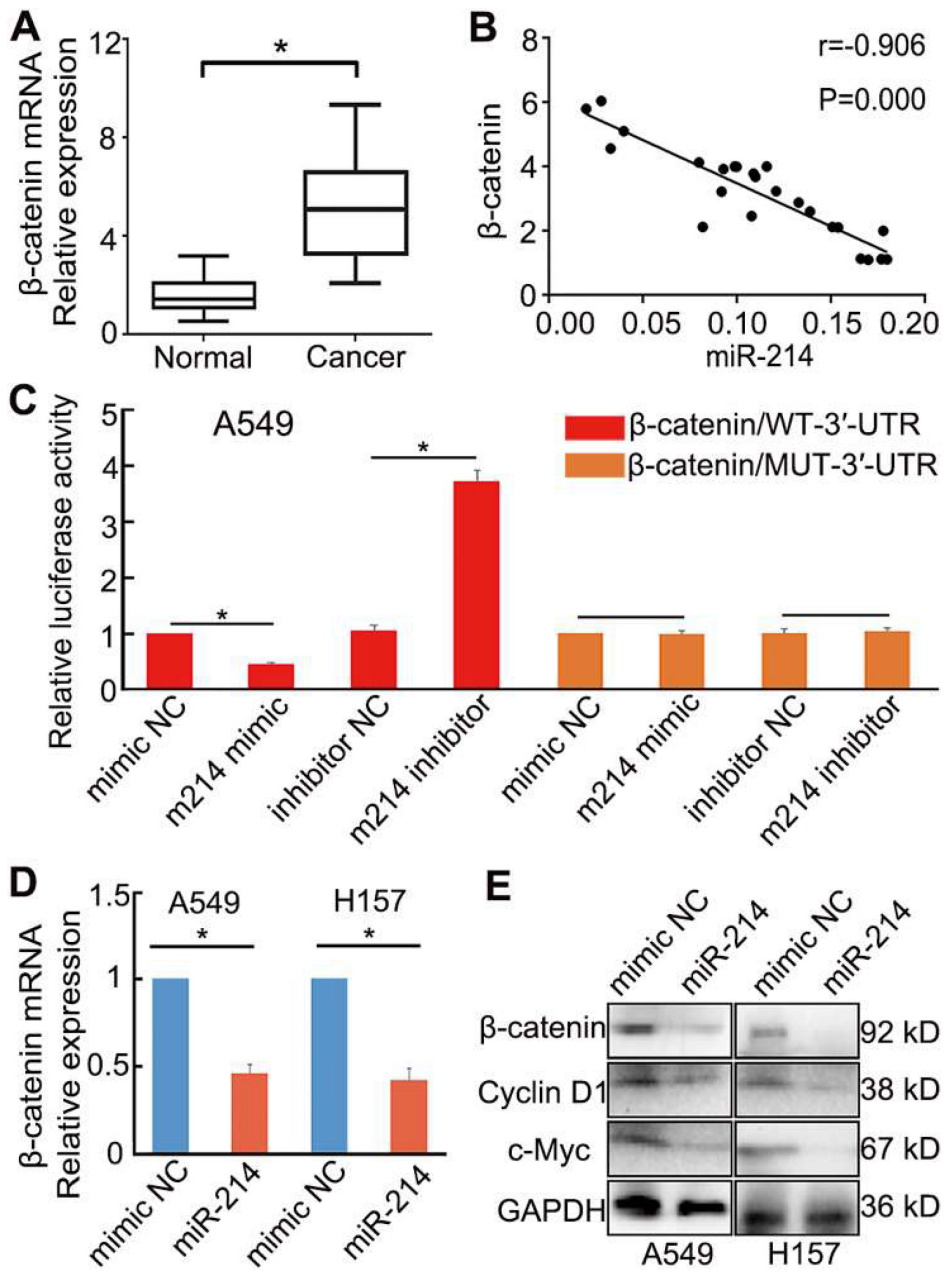
Influence of miR-214 on β-catenin expression in NSCLC cells. (A) β-catenin mRNA expression in NSCLC and paracancerous tissues. (B) The relationship between miR-214 and β-catenin in NSCLC tissues. (C) Dual-luciferase assay analyses of Wnt/β-catenin signalling under different treatments; A549 cells were transfected with β-catenin/WT-3′-UTR or β-catenin/MUT-3′-UTR and then treated with different miRNAs. m214, miR-214. NC, negative control. (D) The effect of miR-214 transfection on β-catenin mRNA expression. (E) The effect of miR-214 transfection on the target genes of Wnt/β-catenin signalling. KD, K Da; *, *P* < 0.05.

To confirm this prediction, we examined the expression of β-catenin in NSCLC and paracancerous tissues. β-catenin expression in NSCLC tissues was upregulated considerably compared with that in paracancerous tissues (*P* < 0.05; Figure 5A). Moreover, the expression of β-catenin was negatively correlated with that of miR-214 in NSCLC tissues, as assessed by qRT-PCR (Figure 5B)*.*

To investigate the role of miR-214 in Wnt/β-catenin signalling in NSCLC, A549 cells were transfected with either an miR-214 mimic or miR-214 inhibitor. The miR-214 mimic significantly inhibited Wnt/β-catenin signalling, mRNA and protein expression levels of β-catenin, and expression of target genes in the Wnt/β-catenin pathway; conversely, the miR-214 inhibitor significantly enhanced these parameters (*P* < 0.05; Figure 5C-E).

A luciferase reporter assay was then applied to detect the putative miR-214-dependent post-transcriptional regulation of *CTNNB1*. A549 cells were co-transfected with the miR-214 mimic or mimic control and β-catenin/mut-3′-UTR (MUT) or β-catenin-3′-UTR (WT). The luciferase activity in the cells transfected with β-catenin-3′-UTR and miR-214 mimic was significantly decreased compared with that in the control; however, cells transfected with the β-catenin/mut-3′-UTR and miR-214 mimic showed no significant changes in activity relative to control cells (Figure 5C).

### Dvl1 reverses miR-214-mediated inhibition of Wnt/β-catenin signalling in NSCLC cells

**Figure 6:**
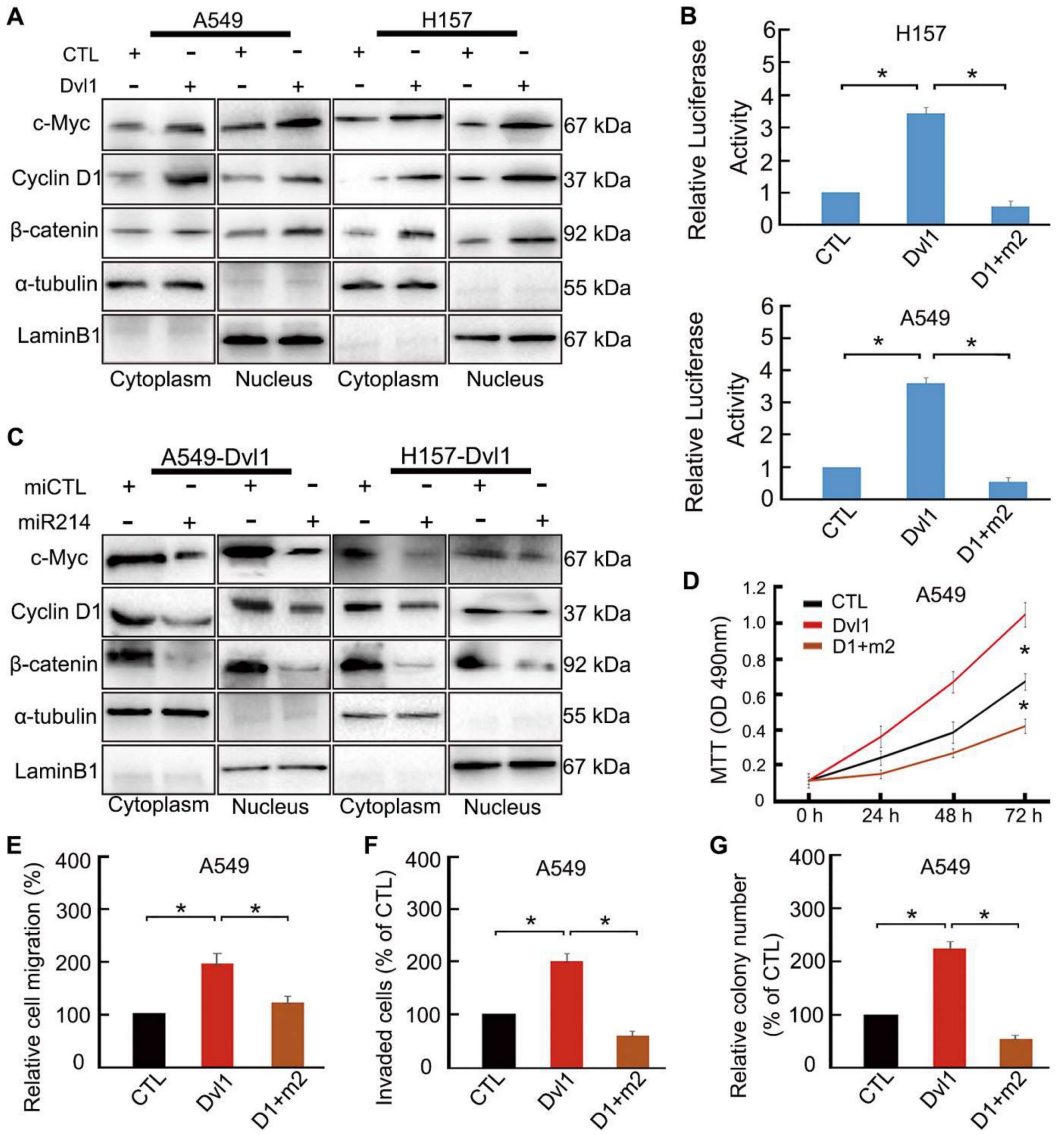
Restoring the effect of Dvl1 on the inhibition of miR-214 in Wnt/β-catenin of NSCLC cells. (A) The effect of Dvl1 transfection on β-catenin nuclear translocation and the expression of Cyclin D1 and c-Myc in A549 and H157 cells. (B) Dual-luciferase assay analyses of Wnt/β-catenin signalling. (C) The effect of Dvl1 and miR-214 co-transfection on β-catenin nuclear translocation and the expression of Cyclin D1 and c-Myc in A549 and H157 cells. The graph shows the influence of Dvl1 and miR-214 on NSCLC (D) cell proliferation, (E) migration, (F) invasiveness, and (G) colony formation of A549 cells. CTL, control, the control group of Dvl1 expression plasmid; Dvl1, Dvl1 expression plasmid transfection. D1+m2, Dvl1 and miR-214 co-transfection. α-tubulin: cytoplasm control. Lamin B1: nuclear control. *, *P* < 0.05.

To confirm the role of miR-214 in Dvl1-induced activation of canonical Wnt signalling, we transfected Dvl1 expression plasmids and miR-214 into NSCLC cells. Dvl1 transfection significantly promoted the nuclear translocation of β-catenin, the luciferase activity of β-catenin, and the expression of c-Myc and Cyclin D1 (Figure 6A, B). Upon confirmation of successful co-transfection of miR-214 and Dvl1, the luciferase activity in the cells co-transfected with Dvl1 and miR-214 was found to be significantly downregulated compared with that in cells transfected with Dvl1 alone (Figure 6B). Furthermore, β-catenin nuclear translocation and Cyclin D1 and c-Myc expression were significantly decreased (*P* < 0.05; Figure 6C), as was the malignant phenotype of A549 cells (Figure 6D-G).

## Discussion

Understanding the molecular mechanisms of NSCLC malignant phenotypes is crucial for achieving a favourable prognosis for patients. Dysregulation of signalling pathways during malignant progression is partly attributable to the aberrant expression of miRNAs 16. miRNAs are small noncoding RNAs that complementarily interact with the 3′-UTR of target mRNAs to degrade or translationally inhibit them 17,18. Nearly 30% of all mRNAs are potential miRNA targets. Each miRNA targets multiple functionally related genes to mediate signalling networks 18,19. Many miRNAs have been implicated in tumorigenesis, functioning as either oncogenes or tumour suppressors. miR-21 affects the biological status, such as invasion, of NSCLC cells through aberrant EGFR signalling 20. Some miRNA alterations and targeting effects have been found in NSCLC. For example, the interaction of miR-4731-5p with ribosomal protein large P0 is crucial for the progression of NSCLC 21. These studies suggest that miRNA participates in lung carcinogenesis.

Interestingly, miR-214 works with β-catenin as a heterotypic transcriptional complex and negatively regulates canonical Wnt downstream gene transcription 22. To identify the possible targets of miR-214 in NSCLC cells, the TargetScan software was used, and the prediction results confirmed that β-catenin is the most likely target of miR-214. Our results confirm that miR-214 directly targets the 3′-UTR of β-catenin to inhibit Wnt/β-catenin signalling in NSCLC cells, suggesting that miR-214 plays a key role in canonical Wnt-β-catenin signalling.

Wnt signalling plays an important role in embryonic development, whose deregulation leads to the initiation and progression of human malignancy via regulation of target gene expression. Canonical Wnt/β-catenin signalling is a potential therapeutic target in malignancies, such as NSCLC and triple-negative breast cancer 23. Malignant tumour cells acquire invasive-associated phenotypes via regulation of the Wnt pathway. Dvl plays an important role in transmitting Wnt signals in development and carcinogenesis in humans. The present study confirmed that Dvl1 promotes NSCLC progression. However, the mechanism of Dvl1 integrating upstream signals and transmitting them to downstream components has so far been poorly understood.

In the Wnt/β-catenin pathway, free cytoplasmic β-catenin is regulated by Wnt signalling. Stable expression of cytoplasmic β-catenin depends on a poly-complex that includes glycogen synthase kinase 3β (GSK-3β), axin, casein kinase Iα, and adenomatous polyposis coli 24,25. β-catenin is phosphorylated and degraded by this complex. When a Wnt signalling protein binds to a cell surface receptor, Dvl proteins inhibit GSK-3β-dependent phosphorylation of β-catenin so that β-catenin is not degraded, leading to its cytoplasmic accumulation. Then, cytoplasmic β-catenin translocates into the nucleus to stimulate the transcription of Wnt downstream target genes. Dvl1-mediated activation of the canonical Wnt/β-catenin pathway is important for the NSCLC malignant phenotype 10. Herein, Dvl1 was found to promote β-catenin nuclear translocation to promote NSCLC progression.

miR-214 is a negative factor for canonical Wnt signalling, whereas Dvl1 is a positive factor. This indicates potential antagonistic effects between them. We found that Dvl1 reverses miR-214-mediated inhibition of Wnt/β-catenin signalling in NSCLC cells. Our results indicate that the interaction of miR-214 with β-catenin inhibits the Dvl1-induced activation of canonical Wnt signalling. In recent years, many databases have been developed, such as TargetScan and PicTar, facilitating bioinformatics analyses. Bioinformatics-based prediction indicates that miR-214 targets β-catenin at the 3′-UTR and regulates its expression. Our luciferase reporter assay confirmed this prediction in NSCLC cells.

miRNAs detected in tissues, either down- or upregulated, are associated with various diseases. Takamizawa et al. discovered that the reduced expression of *let-7* miRNA was associated with poor prognosis in NSCLC 26. Likewise, miR-184 and miR-489-3p functionally converge on canonical Wnt signalling for NSCLC diagnosis and drug resistance prediction 27. Whether these miRNAs are involved in the Dvl1-induced activation of canonical Wnt/β-catenin pathway and whether one or more of them work in synergy with miR-214 remains to be explored in the future.

In summary, we confirmed that miR-214 binds to the 3′-UTR of β-catenin. Dvl1 activates canonical Wnt signalling by inhibiting the interaction of miR-214 with β-catenin and promoting stable β-catenin nuclear translocation in NSCLC cells, which contributes to pulmonary tumorigenesis. We showed that miR-214 is not only sufficient but also required for Dvl1-induced activation of the Wnt/β-catenin pathway, and the interaction between miR-214 and β-catenin is critical for this process. Notably, the inhibition of miR-214 and β-catenin interaction is critical for promoting Dvl1-induced β-catenin nuclear translocation and canonical Wnt signalling activation. Therefore, our results suggest that Dvl1 is a potential therapeutic target for NSCLC and that miR-214 plays an inhibitory role in Dvl1-mediated activation of Wnt/β-catenin signalling in NSCLC cells, which may affect the progression of NSCLC.

## Supplementary Material

Supplementary figures and table.

## Figures and Tables

**Table 1:** Dvl1 expression in relation to clinicopathological variables

Variables	Total	Dvl1 expression		*P*
		Negative	Positive	
	122	47	75	
*Age (years)*				
<60	45	16	29	0.606
≥60	77	31	46	
*Sex*				
Male	71	31	40	0.169
Female	51	16	35	
*Histologic type*				
SCC	47	24	23	0.024
AC	75	23	52	
*Differentiation*				
Well	46	26	20	0.001
Moderate or poor	76	21	55	
*Lymphatic metastasis*				
No	52	24	28	0.136
Yes	70	23	47	
*pTNM stage*				
I	44	25	19	0.002
II+III	78	22	56	
*β-catenin*				
Negative	33	18	15	0.027
Positive	89	29	60	
SCC, squamous cell carcinoma; AC, adenocarcinoma.				

**Table 2:** Relationship between Dvl1 and β-catenin in NSCLC

	Dvl1					
β-catenin	Cyt-coexpression		Nuc-coexpression		Mem-coexpresion	
	No	Yes	No	Yes	No	Yes
No	50	15	105	2	94	11
Yes	25	32	2	13	15	2
Total	75	47	107	15	109	13

Cyt: cytoplasmic; Nuc: nuclear; Mem: membranous

## Abbreviations

Dvl: dishevelled
LEF: lymphoid enhancer factor
microRNA: miRNA
microRNA-214: miR-214
NSCLC: non-small cell lung cancer
qRT-PCR: quantitative real-time polymerase chain reaction
TCF: T-cell factor
3'-untranslated region: 3'-UTR
