# Supplementary material for: Role of MicroRNA-214 in Dishevelled1-Modulated β-catenin Signalling in Non-Small Cell Lung Cancer Progression
Source: J Cancer. 2023 Jan 1;14(2):239–49. doi: 10.7150/jca.80291 (PMC9891876; doi:10.7150/jca.80291)
Supplement: Supplementary file 1 — Supplementary figures and table. [file jcav14p0239s1.pdf]

# *Supplementary Material*

## Supplementary Table

**Table S1.** Sequences of related primers.

| Factor | Forward | Reverse |
|---|---|---|
| Dvl1 | 5'-CCCCTCCTTCCACCCAAATG-3' | 5'-GTGACTGACCATGGACTCCG-3' |
| β-catenin | 5'-CAGCGACTAAGCAGGAAG-3' | 5'-GATGACGAAGAGCACAGAT-3' |
| β-actin | 5'-AGAAGGCTGGGGCTCATTTG-3' | 5'-AGGGGCCATCCACAGTCTTC-3' |
| miR-214 | 5'-GGACAGGACGCACAGTCA-3' | 5'-CAGACGAGGCTCCGTGGT-3' |
| U6 | 5’-CTCGCTTCGGCAGCACA-3’ | 5’-AACGCTTCACGAATTTGCGT-3’ |

**Supplementary Figures**

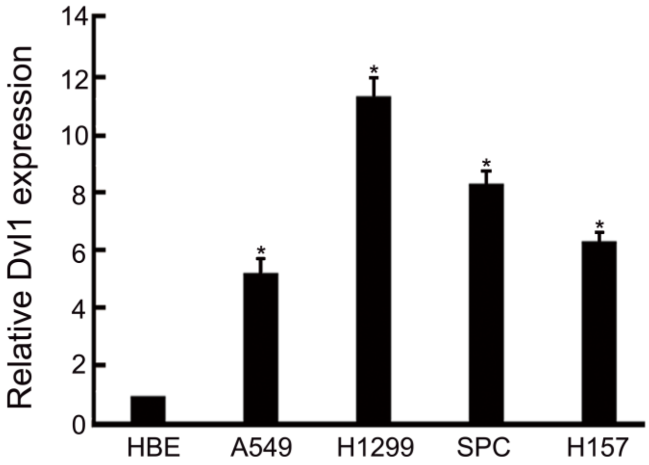


**Figure S1**. mRNA expression of Dvl1 in NSCLC cell lines.

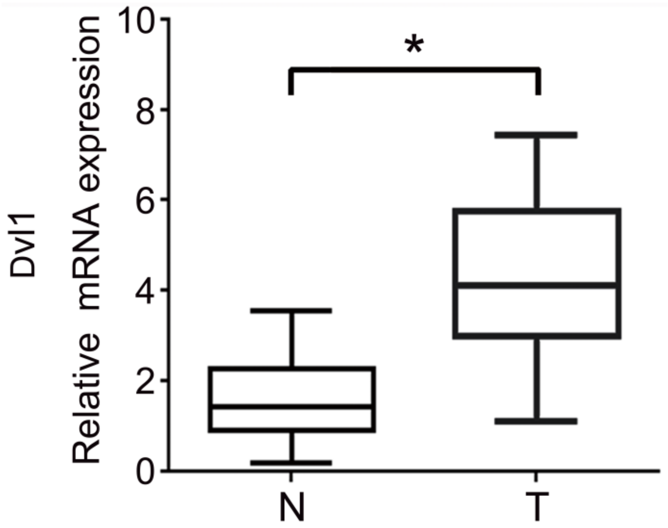


**Figure S2**. mRNA expression of Dvl1 in NSCLC tissues (T) and paired normal lung tissues (N).

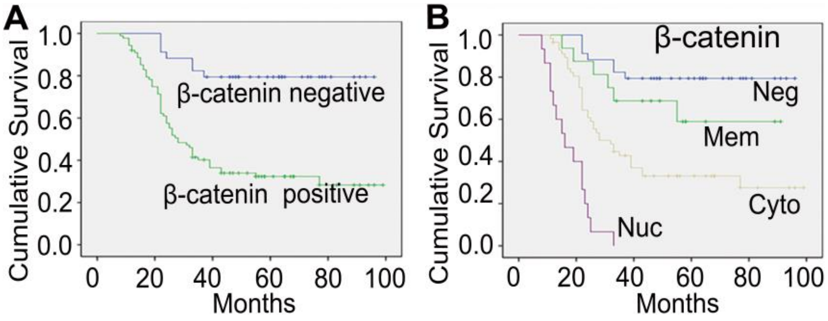


**Figure S3.** Kaplan–Meier curves of overall survival among patients with NSCLC. (A) The groups with β-catenin-positive expression and negative expression; (B) the groups with β-catenin-negative expression (Neg), positive membranous expression (Mem), positive cytoplasmic expression (Cyto), and positive nuclear expression (Nuc).

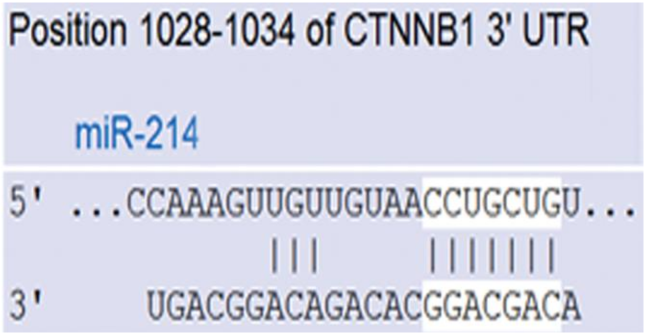


**Figure S4**. Bioinformatics analysis using the target prediction software TargetScan.
